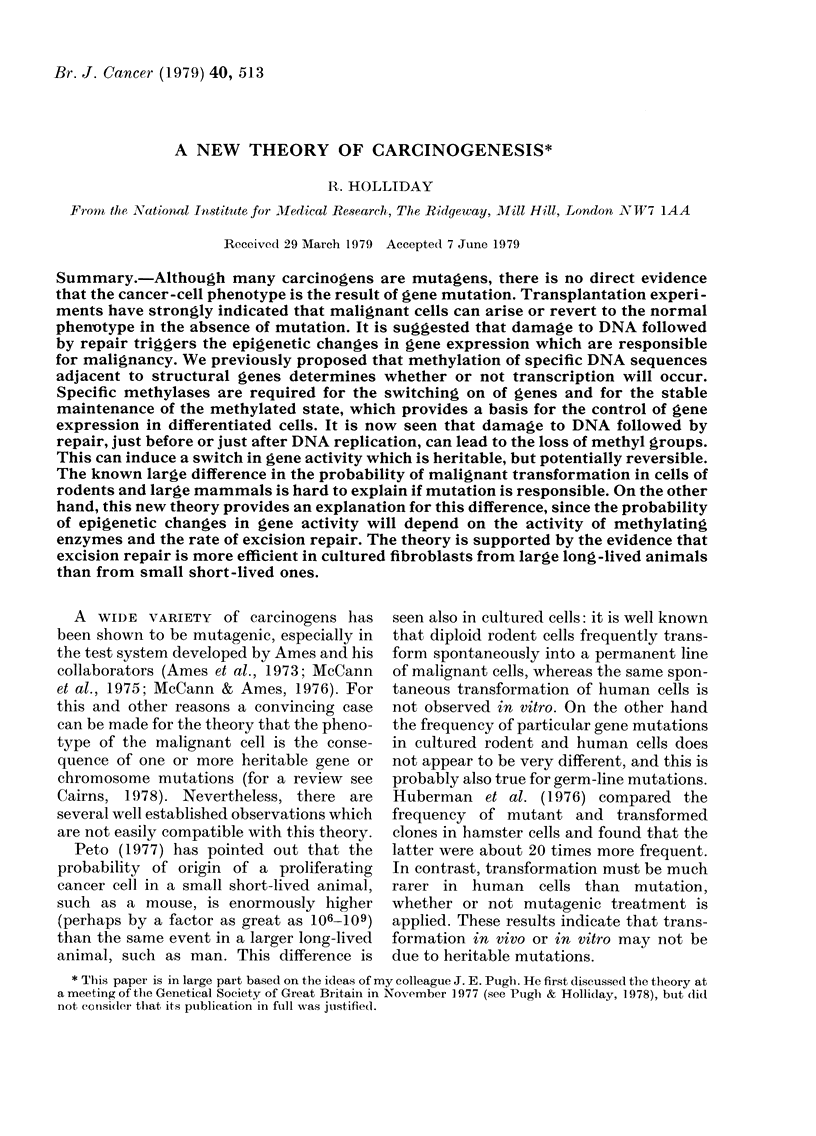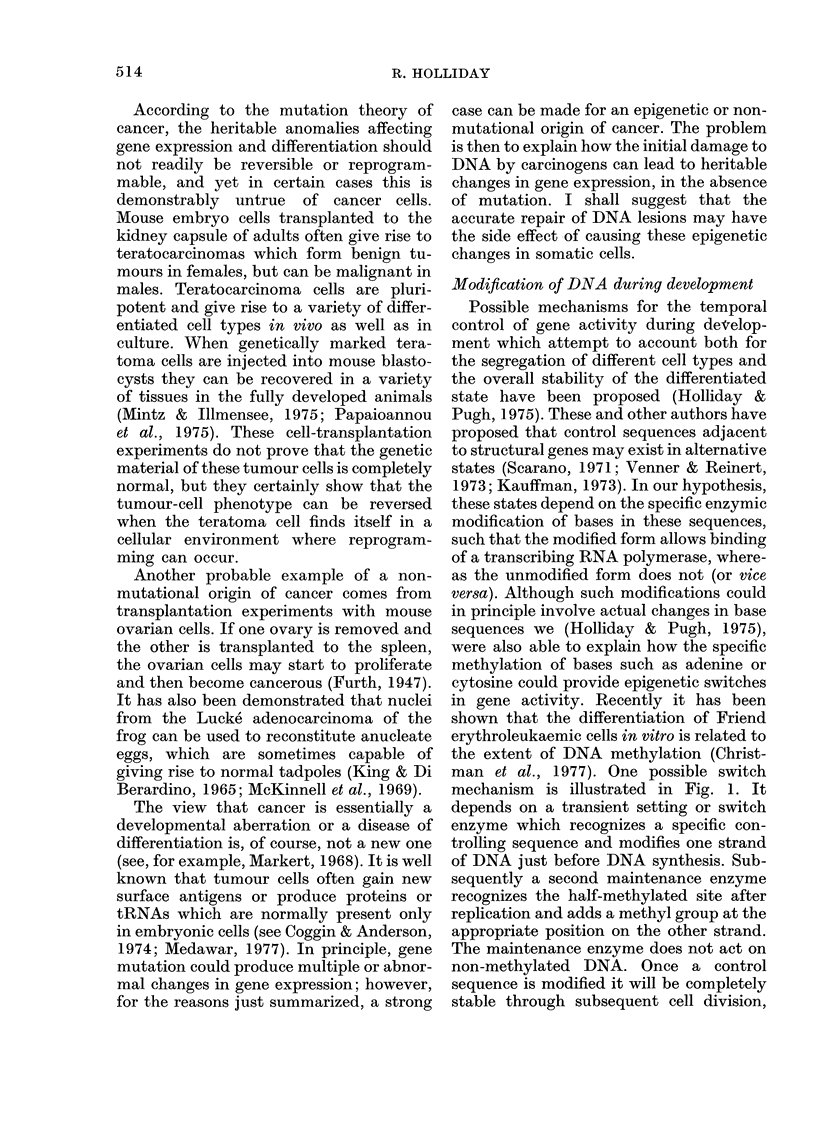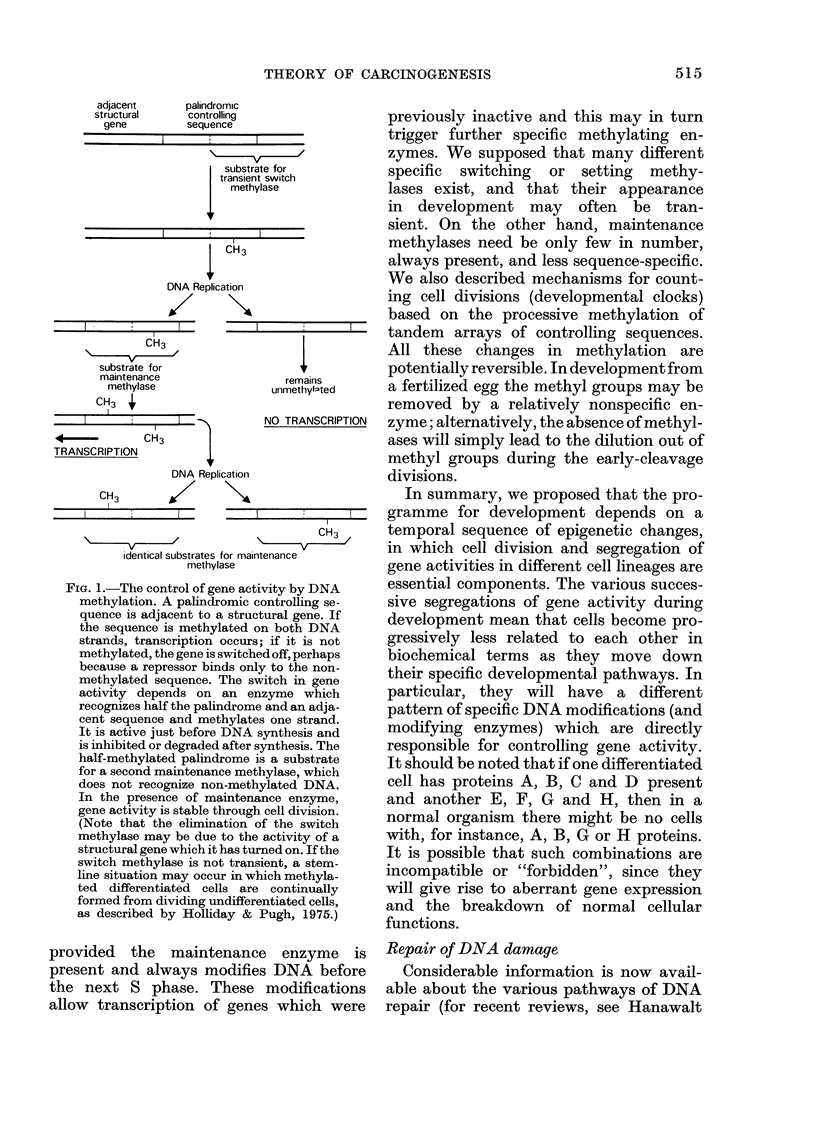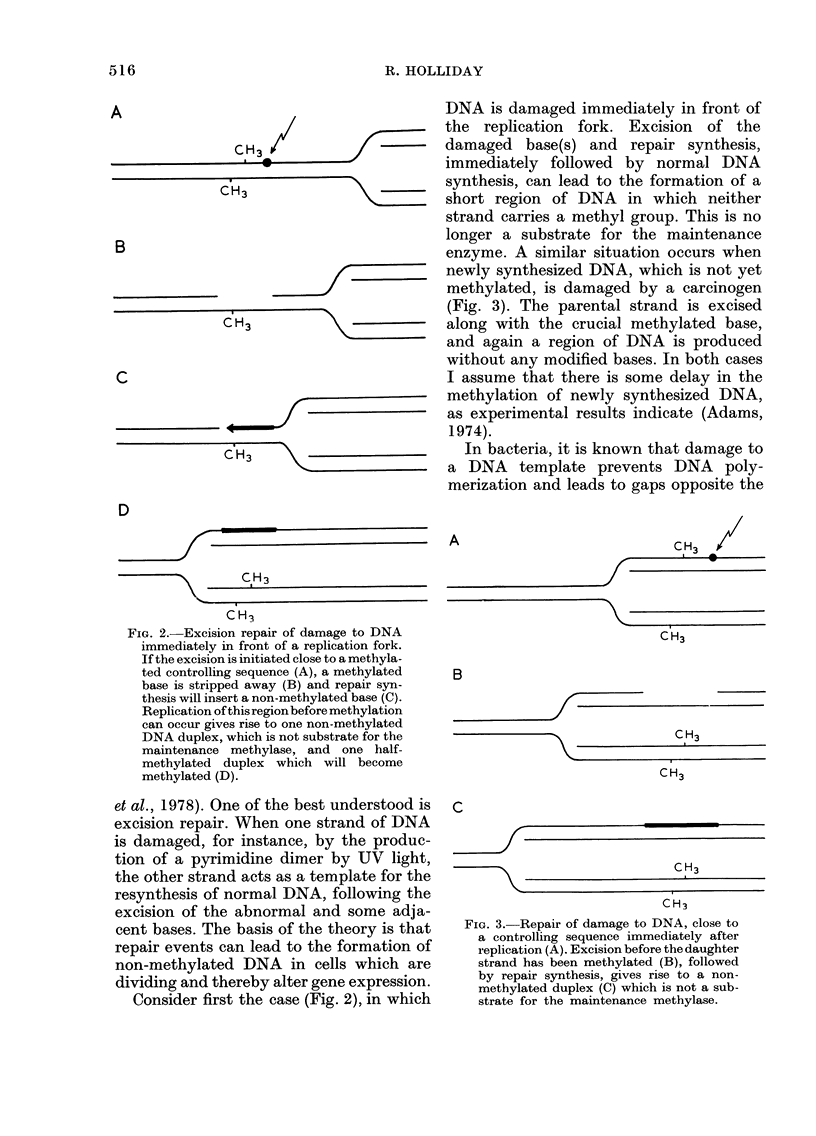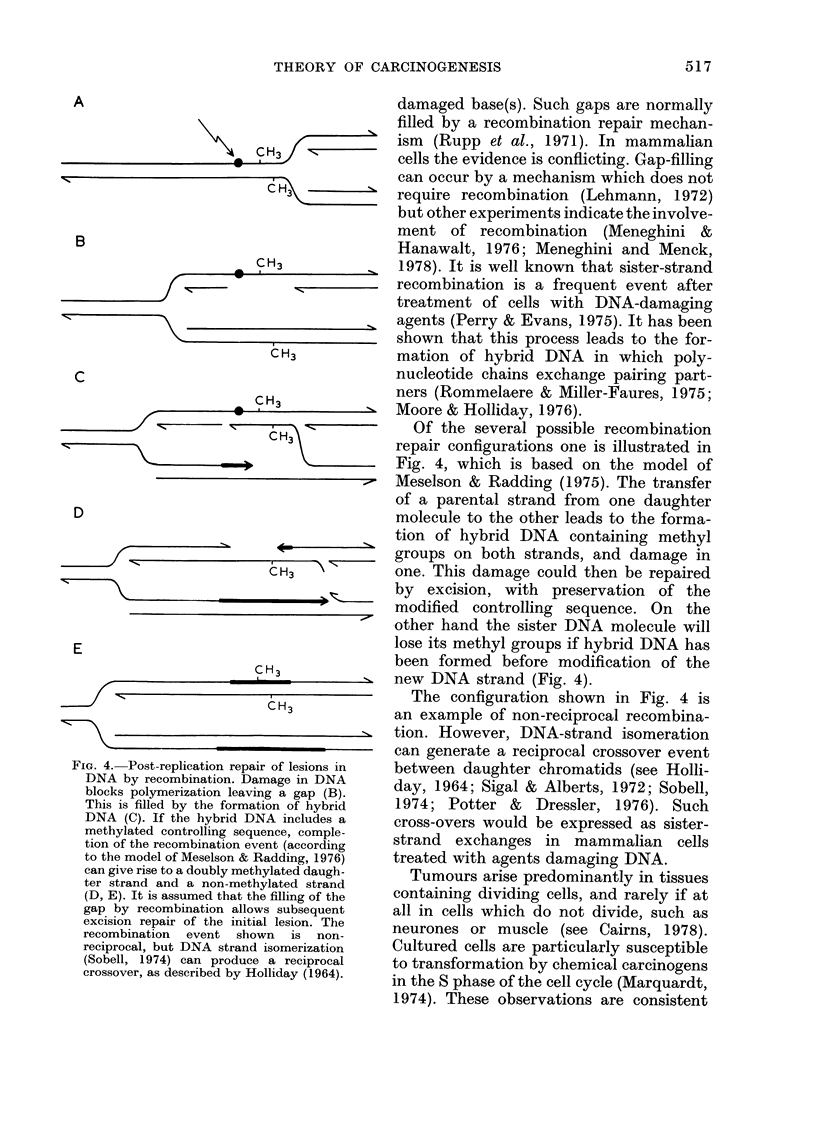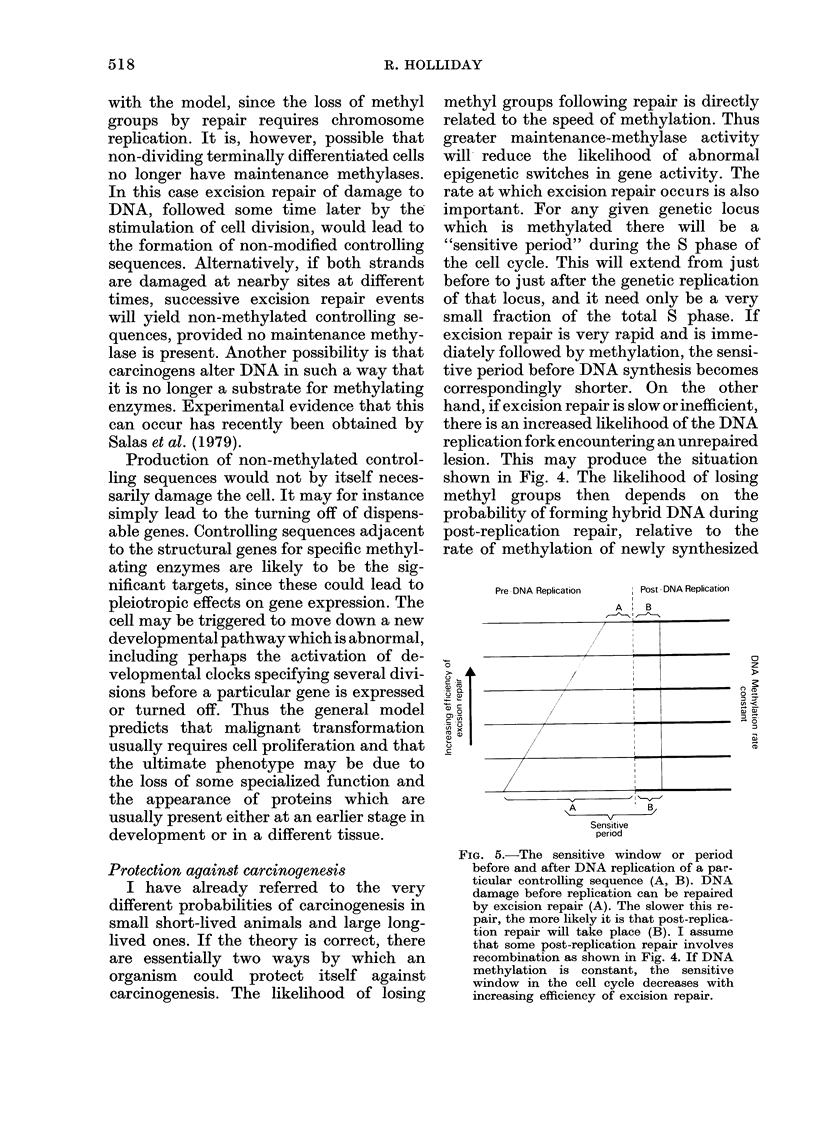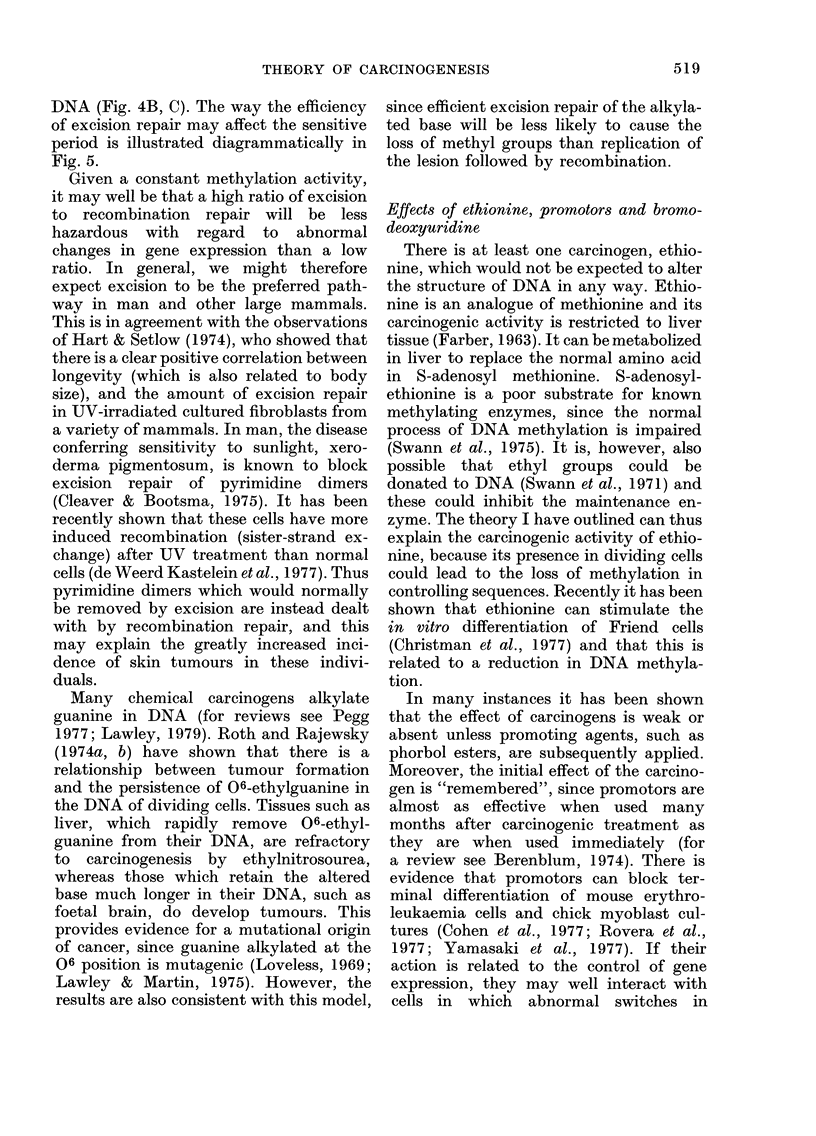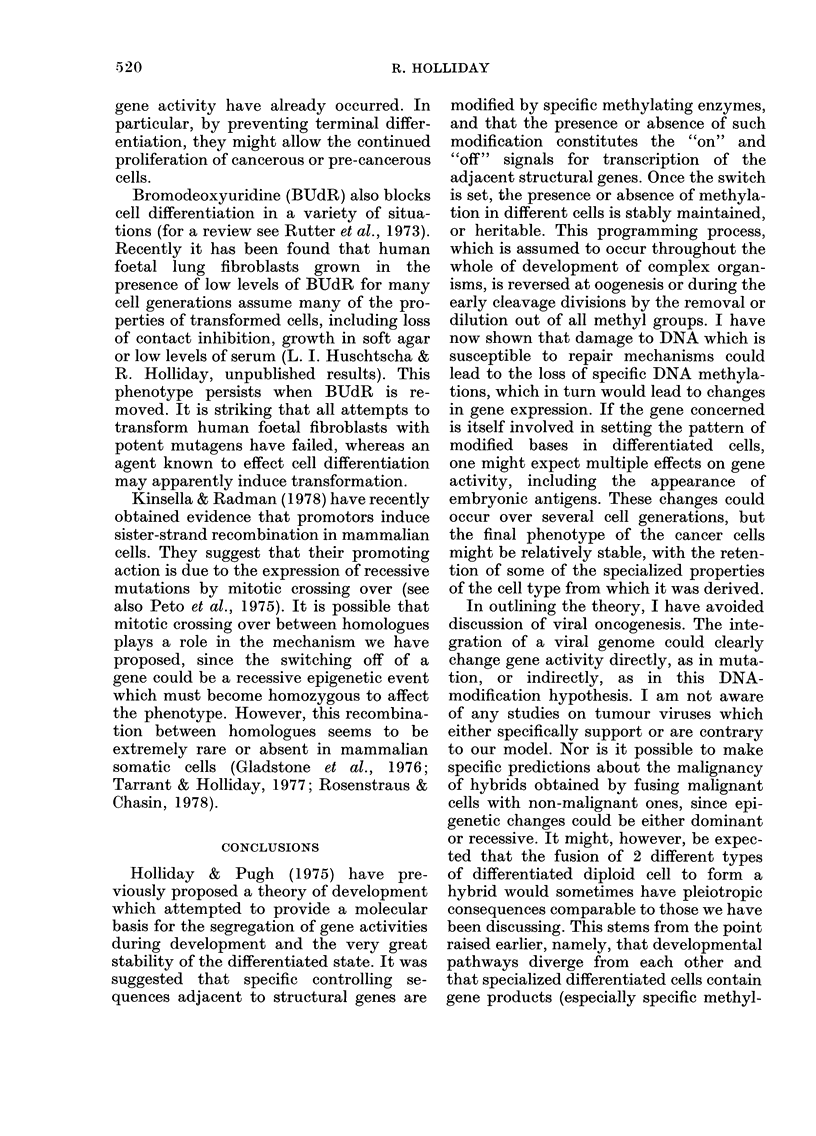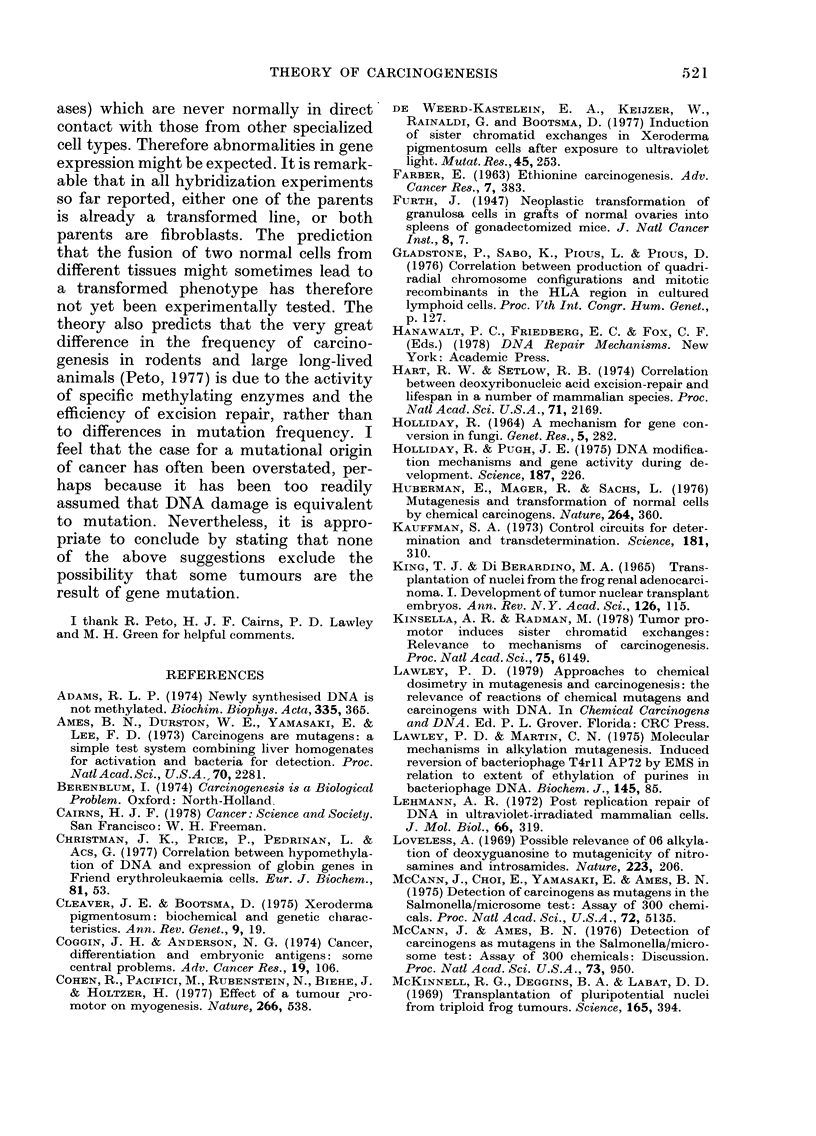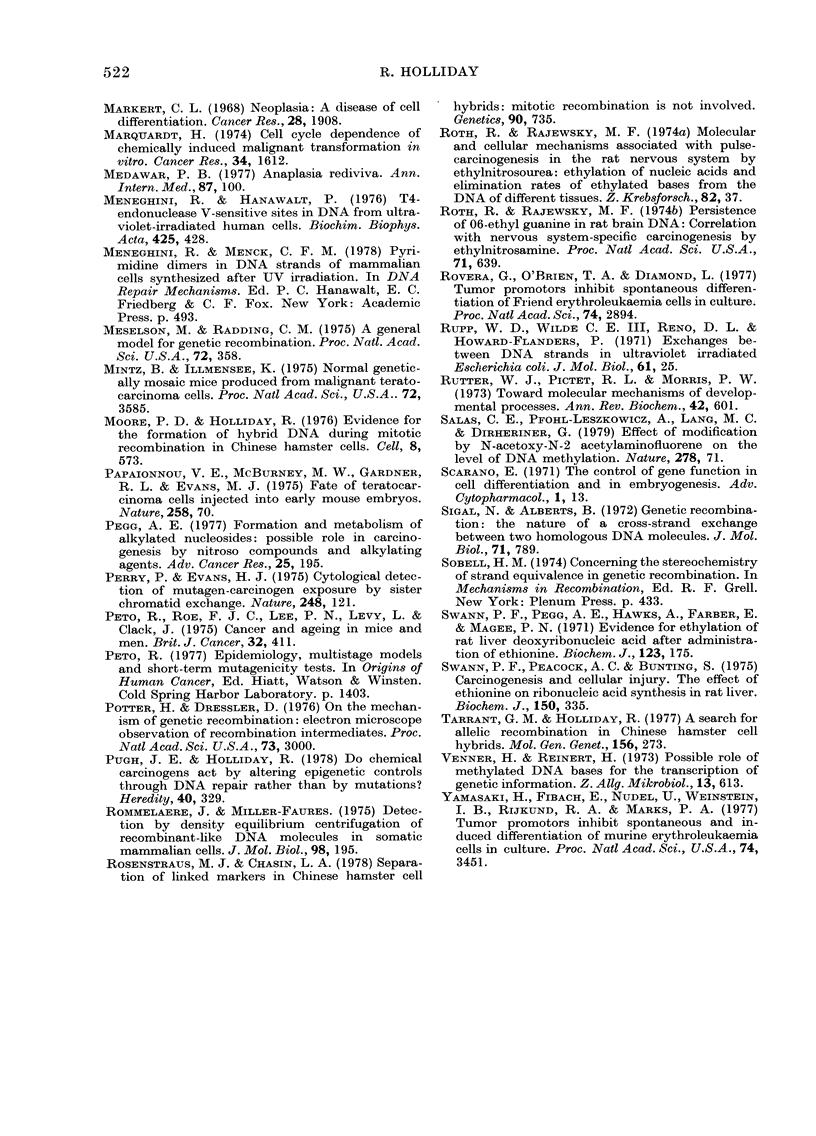# A new theory of carcinogenesis*

**DOI:** 10.1038/bjc.1979.216

**Published:** 1979-10

**Authors:** R. Holliday

## Abstract

Although many carcinogens are mutagens, there is no direct evidence that the cancer-cell phenotype is the result of gene mutation. Transplantation experiments have strongly indicated that malignant cells can arise or revert to the normal phenotype in the absence of mutation. It is suggested that damage to DNA followed by repair triggers the epigenetic changes in gene expression which are responsible for malignancy. We previously proposed that methylation of specific DNA sequences adjacent to structural genes determines whether or not transcription will occur. Specific methylases are required for the switching on of genes and for the stable maintenance of the methylated state, which provides a basis for the control of gene expression in differentiated cells. It is now seen that damage to DNA followed by repair, just before or just after DNA replication, can lead to the loss of methyl groups. This can induce a switch in gene activity which is heritable, but potentially reversible. The known large difference in the probability of malignant transformation in cells of rodents and large mammals is hard to explain if mutation is responsible. On the other hand, this new theory provides an explanation for this difference, since the probability of epigenetic changes in gene activity will depend on the activity of methylating enzymes and the rate of excision repair. The theory is supported by the evidence that excision repair is more efficient in cultured fibroblasts from large long-lived animals than from small short-lived ones.


*Br. J. Cancer* (1979) **40**, 513

### A NEW THEORY OF CARCINOGENESIS*

R. HOLLIDAY

*From the National Institute for Medical Research, The Ridgeway, Mill Hill, London NW7 1AA*

Received 29 March 1979 Accepted 7 June 1979

**Summary.—Although many carcinogens are mutagens, there is no direct evidence that the cancer-cell phenotype is the result of gene mutation. Transplantation experiments have strongly indicated that malignant cells can arise or revert to the normal phenotype in the absence of mutation. It is suggested that damage to DNA followed by repair triggers the epigenetic changes in gene expression which are responsible for malignancy. We previously proposed that methylation of specific DNA sequences adjacent to structural genes determines whether or not transcription will occur. Specific methylases are required for the switching on of genes and for the stable maintenance of the methylated state, which provides a basis for the control of gene expression in differentiated cells. It is now seen that damage to DNA followed by repair, just before or just after DNA replication, can lead to the loss of methyl groups. This can induce a switch in gene activity which is heritable, but potentially reversible. The known large difference in the probability of malignant transformation in cells of rodents and large mammals is hard to explain if mutation is responsible. On the other hand, this new theory provides an explanation for this difference, since the probability of epigenetic changes in gene activity will depend on the activity of methylating enzymes and the rate of excision repair. The theory is supported by the evidence that excision repair is more efficient in cultured fibroblasts from large long-lived animals than from small short-lived ones.**

A WIDE VARIETY of carcinogens has been shown to be mutagenic, especially in the test system developed by Ames and his collaborators (Ames *et al.*, 1973; McCann *et al.*, 1975; McCann & Ames, 1976). For this and other reasons a convincing case can be made for the theory that the phenotype of the malignant cell is the consequence of one or more heritable gene or chromosome mutations (for a review see Cairns, 1978). Nevertheless, there are several well established observations which are not easily compatible with this theory.

Peto (1977) has pointed out that the probability of origin of a proliferating cancer cell in a small short-lived animal, such as a mouse, is enormously higher (perhaps by a factor as great as $10^6$–$10^9$) than the same event in a larger long-lived animal, such as man. This difference is seen also in cultured cells: it is well known that diploid rodent cells frequently transform spontaneously into a permanent line of malignant cells, whereas the same spontaneous transformation of human cells is not observed *in vitro*. On the other hand the frequency of particular gene mutations in cultured rodent and human cells does not appear to be very different, and this is probably also true for germ-line mutations. Huberman *et al.* (1976) compared the frequency of mutant and transformed clones in hamster cells and found that the latter were about 20 times more frequent. In contrast, transformation must be much rarer in human cells than mutation, whether or not mutagenic treatment is applied. These results indicate that transformation *in vivo* or *in vitro* may not be due to heritable mutations.

* This paper is in large part based on the ideas of my colleague J. E. Pugh. He first discussed the theory at a meeting of the Genetical Society of Great Britain in November 1977 (see Pugh & Holliday, 1978), but did not consider that its publication in full was justified.

According to the mutation theory of cancer, the heritable anomalies affecting gene expression and differentiation should not readily be reversible or reprogrammable, and yet in certain cases this is demonstrably untrue of cancer cells. Mouse embryo cells transplanted to the kidney capsule of adults often give rise to teratocarcinomas which form benign tumours in females, but can be malignant in males. Teratocarcinoma cells are pluripotent and give rise to a variety of differentiated cell types *in vivo* as well as in culture. When genetically marked teratoma cells are injected into mouse blastocysts they can be recovered in a variety of tissues in the fully developed animals (Mintz & Illmensee, 1975; Papaioannou *et al.*, 1975). These cell-transplantation experiments do not prove that the genetic material of these tumour cells is completely normal, but they certainly show that the tumour-cell phenotype can be reversed when the teratoma cell finds itself in a cellular environment where reprogramming can occur.

Another probable example of a non-mutational origin of cancer comes from transplantation experiments with mouse ovarian cells. If one ovary is removed and the other is transplanted to the spleen, the ovarian cells may start to proliferate and then become cancerous (Furth, 1947). It has also been demonstrated that nuclei from the Lucké adenocarcinoma of the frog can be used to reconstitute anucleate eggs, which are sometimes capable of giving rise to normal tadpoles (King & Di Berardino, 1965; McKinnell *et al.*, 1969).

The view that cancer is essentially a developmental aberration or a disease of differentiation is, of course, not a new one (see, for example, Markert, 1968). It is well known that tumour cells often gain new surface antigens or produce proteins or tRNAs which are normally present only in embryonic cells (see Coggin & Anderson, 1974; Medawar, 1977). In principle, gene mutation could produce multiple or abnormal changes in gene expression; however, for the reasons just summarized, a strong case can be made for an epigenetic or non-mutational origin of cancer. The problem is then to explain how the initial damage to DNA by carcinogens can lead to heritable changes in gene expression, in the absence of mutation. I shall suggest that the accurate repair of DNA lesions may have the side effect of causing these epigenetic changes in somatic cells.

*Modification of DNA during development*

Possible mechanisms for the temporal control of gene activity during development which attempt to account both for the segregation of different cell types and the overall stability of the differentiated state have been proposed (Holliday & Pugh, 1975). These and other authors have proposed that control sequences adjacent to structural genes may exist in alternative states (Scarano, 1971; Venner & Reinert, 1973; Kauffman, 1973). In our hypothesis, these states depend on the specific enzymic modification of bases in these sequences, such that the modified form allows binding of a transcribing RNA polymerase, whereas the unmodified form does not (or *vice versa*). Although such modifications could in principle involve actual changes in base sequences we (Holliday & Pugh, 1975), were also able to explain how the specific methylation of bases such as adenine or cytosine could provide epigenetic switches in gene activity. Recently it has been shown that the differentiation of Friend erythroleukaemic cells *in vitro* is related to the extent of DNA methylation (Christman *et al.*, 1977). One possible switch mechanism is illustrated in Fig. 1. It depends on a transient setting or switch enzyme which recognizes a specific controlling sequence and modifies one strand of DNA just before DNA synthesis. Subsequently a second maintenance enzyme recognizes the half-methylated site after replication and adds a methyl group at the appropriate position on the other strand. The maintenance enzyme does not act on non-methylated DNA. Once a control sequence is modified it will be completely stable through subsequent cell division,

FIG. 1.—The control of gene activity by DNA methylation. A palindromic controlling sequence is adjacent to a structural gene. If the sequence is methylated on both DNA strands, transcription occurs; if it is not methylated, the gene is switched off, perhaps because a repressor binds only to the non-methylated sequence. The switch in gene activity depends on an enzyme which recognizes half the palindrome and an adjacent sequence and methylates one strand. It is active just before DNA synthesis and is inhibited or degraded after synthesis. The half-methylated palindrome is a substrate for a second maintenance methylase, which does not recognize non-methylated DNA. In the presence of maintenance enzyme, gene activity is stable through cell division. (Note that the elimination of the switch methylase may be due to the activity of a structural gene which it has turned on. If the switch methylase is not transient, a stem-line situation may occur in which methylated differentiated cells are continually formed from dividing undifferentiated cells, as described by Holliday & Pugh, 1975.)

provided the maintenance enzyme is present and always modifies DNA before the next S phase. These modifications allow transcription of genes which were previously inactive and this may in turn trigger further specific methylating enzymes. We supposed that many different specific switching or setting methylases exist, and that their appearance in development may often be transient. On the other hand, maintenance methylases need be only few in number, always present, and less sequence-specific. We also described mechanisms for counting cell divisions (developmental clocks) based on the processive methylation of tandem arrays of controlling sequences. All these changes in methylation are potentially reversible. In development from a fertilized egg the methyl groups may be removed by a relatively nonspecific enzyme; alternatively, the absence of methylases will simply lead to the dilution out of methyl groups during the early-cleavage divisions.

In summary, we proposed that the programme for development depends on a temporal sequence of epigenetic changes, in which cell division and segregation of gene activities in different cell lineages are essential components. The various successive segregations of gene activity during development mean that cells become progressively less related to each other in biochemical terms as they move down their specific developmental pathways. In particular, they will have a different pattern of specific DNA modifications (and modifying enzymes) which are directly responsible for controlling gene activity. It should be noted that if one differentiated cell has proteins A, B, C and D present and another E, F, G and H, then in a normal organism there might be no cells with, for instance, A, B, G or H proteins. It is possible that such combinations are incompatible or "forbidden", since they will give rise to aberrant gene expression and the breakdown of normal cellular functions.

*Repair of DNA damage*

Considerable information is now available about the various pathways of DNA repair (for recent reviews, see Hanawalt

FIG. 2.—Excision repair of damage to DNA immediately in front of a replication fork. If the excision is initiated close to a methylated controlling sequence (A), a methylated base is stripped away (B) and repair synthesis will insert a non-methylated base (C). Replication of this region before methylation can occur gives rise to one non-methylated DNA duplex, which is not substrate for the maintenance methylase, and one half-methylated duplex which will become methylated (D).

*et al.*, 1978). One of the best understood is excision repair. When one strand of DNA is damaged, for instance, by the production of a pyrimidine dimer by UV light, the other strand acts as a template for the resynthesis of normal DNA, following the excision of the abnormal and some adjacent bases. The basis of the theory is that repair events can lead to the formation of non-methylated DNA in cells which are dividing and thereby alter gene expression.

Consider first the case (Fig. 2), in which DNA is damaged immediately in front of the replication fork. Excision of the damaged base(s) and repair synthesis, immediately followed by normal DNA synthesis, can lead to the formation of a short region of DNA in which neither strand carries a methyl group. This is no longer a substrate for the maintenance enzyme. A similar situation occurs when newly synthesized DNA, which is not yet methylated, is damaged by a carcinogen (Fig. 3). The parental strand is excised along with the crucial methylated base, and again a region of DNA is produced without any modified bases. In both cases I assume that there is some delay in the methylation of newly synthesized DNA, as experimental results indicate (Adams, 1974).

In bacteria, it is known that damage to a DNA template prevents DNA polymerization and leads to gaps opposite the

FIG. 3.—Repair of damage to DNA, close to a controlling sequence immediately after replication (A). Excision before the daughter strand has been methylated (B), followed by repair synthesis, gives rise to a non-methylated duplex (C) which is not a substrate for the maintenance methylase.

FIG. 4.—Post-replication repair of lesions in DNA by recombination. Damage in DNA blocks polymerization leaving a gap (B). This is filled by the formation of hybrid DNA (C). If the hybrid DNA includes a methylated controlling sequence, completion of the recombination event (according to the model of Meselson & Radding, 1976) can give rise to a doubly methylated daughter strand and a non-methylated strand (D, E). It is assumed that the filling of the gap by recombination allows subsequent excision repair of the initial lesion. The recombination event shown is non-reciprocal, but DNA strand isomerization (Sobell, 1974) can produce a reciprocal crossover, as described by Holliday (1964).

damaged base(s). Such gaps are normally filled by a recombination repair mechanism (Rupp *et al.*, 1971). In mammalian cells the evidence is conflicting. Gap-filling can occur by a mechanism which does not require recombination (Lehmann, 1972) but other experiments indicate the involvement of recombination (Meneghini & Hanawalt, 1976; Meneghini and Menck, 1978). It is well known that sister-strand recombination is a frequent event after treatment of cells with DNA-damaging agents (Perry & Evans, 1975). It has been shown that this process leads to the formation of hybrid DNA in which polynucleotide chains exchange pairing partners (Rommelaere & Miller-Faures, 1975; Moore & Holliday, 1976).

Of the several possible recombination repair configurations one is illustrated in Fig. 4, which is based on the model of Meselson & Radding (1975). The transfer of a parental strand from one daughter molecule to the other leads to the formation of hybrid DNA containing methyl groups on both strands, and damage in one. This damage could then be repaired by excision, with preservation of the modified controlling sequence. On the other hand the sister DNA molecule will lose its methyl groups if hybrid DNA has been formed before modification of the new DNA strand (Fig. 4).

The configuration shown in Fig. 4 is an example of non-reciprocal recombination. However, DNA-strand isomeration can generate a reciprocal crossover event between daughter chromatids (see Holliday, 1964; Sigal & Alberts, 1972; Sobell, 1974; Potter & Dressler, 1976). Such cross-overs would be expressed as sister-strand exchanges in mammalian cells treated with agents damaging DNA.

Tumours arise predominantly in tissues containing dividing cells, and rarely if at all in cells which do not divide, such as neurones or muscle (see Cairns, 1978). Cultured cells are particularly susceptible to transformation by chemical carcinogens in the S phase of the cell cycle (Marquardt, 1974). These observations are consistent with the model, since the loss of methyl groups by repair requires chromosome replication. It is, however, possible that non-dividing terminally differentiated cells no longer have maintenance methylases. In this case excision repair of damage to DNA, followed some time later by the stimulation of cell division, would lead to the formation of non-modified controlling sequences. Alternatively, if both strands are damaged at nearby sites at different times, successive excision repair events will yield non-methylated controlling sequences, provided no maintenance methylase is present. Another possibility is that carcinogens alter DNA in such a way that it is no longer a substrate for methylating enzymes. Experimental evidence that this can occur has recently been obtained by Salas *et al.* (1979).

Production of non-methylated controlling sequences would not by itself necessarily damage the cell. It may for instance simply lead to the turning off of dispensable genes. Controlling sequences adjacent to the structural genes for specific methylating enzymes are likely to be the significant targets, since these could lead to pleiotropic effects on gene expression. The cell may be triggered to move down a new developmental pathway which is abnormal, including perhaps the activation of developmental clocks specifying several divisions before a particular gene is expressed or turned off. Thus the general model predicts that malignant transformation usually requires cell proliferation and that the ultimate phenotype may be due to the loss of some specialized function and the appearance of proteins which are usually present either at an earlier stage in development or in a different tissue.

*Protection against carcinogenesis*

I have already referred to the very different probabilities of carcinogenesis in small short-lived animals and large long-lived ones. If the theory is correct, there are essentially two ways by which an organism could protect itself against carcinogenesis. The likelihood of losing methyl groups following repair is directly related to the speed of methylation. Thus greater maintenance-methylase activity will reduce the likelihood of abnormal epigenetic switches in gene activity. The rate at which excision repair occurs is also important. For any given genetic locus which is methylated there will be a "sensitive period" during the S phase of the cell cycle. This will extend from just before to just after the genetic replication of that locus, and it need only be a very small fraction of the total S phase. If excision repair is very rapid and is immediately followed by methylation, the sensitive period before DNA synthesis becomes correspondingly shorter. On the other hand, if excision repair is slow or inefficient, there is an increased likelihood of the DNA replication fork encountering an unrepaired lesion. This may produce the situation shown in Fig. 4. The likelihood of losing methyl groups then depends on the probability of forming hybrid DNA during post-replication repair, relative to the rate of methylation of newly synthesized

FIG. 5.—The sensitive window or period before and after DNA replication of a particular controlling sequence (A, B). DNA damage before replication can be repaired by excision repair (A). The slower this repair, the more likely it is that post-replication repair will take place (B). I assume that some post-replication repair involves recombination as shown in Fig. 4. If DNA methylation is constant, the sensitive window in the cell cycle decreases with increasing efficiency of excision repair.

DNA (Fig. 4B, C). The way the efficiency of excision repair may affect the sensitive period is illustrated diagrammatically in Fig. 5.

Given a constant methylation activity, it may well be that a high ratio of excision to recombination repair will be less hazardous with regard to abnormal changes in gene expression than a low ratio. In general, we might therefore expect excision to be the preferred pathway in man and other large mammals. This is in agreement with the observations of Hart & Setlow (1974), who showed that there is a clear positive correlation between longevity (which is also related to body size), and the amount of excision repair in UV-irradiated cultured fibroblasts from a variety of mammals. In man, the disease conferring sensitivity to sunlight, xeroderma pigmentosum, is known to block excision repair of pyrimidine dimers (Cleaver & Bootsma, 1975). It has been recently shown that these cells have more induced recombination (sister-strand exchange) after UV treatment than normal cells (de Weerd Kastelein *et al.*, 1977). Thus pyrimidine dimers which would normally be removed by excision are instead dealt with by recombination repair, and this may explain the greatly increased incidence of skin tumours in these individuals.

Many chemical carcinogens alkylate guanine in DNA (for reviews see Pegg 1977; Lawley, 1979). Roth and Rajewsky (1974*a*, *b*) have shown that there is a relationship between tumour formation and the persistence of $O^6$-ethylguanine in the DNA of dividing cells. Tissues such as liver, which rapidly remove $O^6$-ethylguanine from their DNA, are refractory to carcinogenesis by ethylnitrosourea, whereas those which retain the altered base much longer in their DNA, such as foetal brain, do develop tumours. This provides evidence for a mutational origin of cancer, since guanine alkylated at the $O^6$ position is mutagenic (Loveless, 1969; Lawley & Martin, 1975). However, the results are also consistent with this model, since efficient excision repair of the alkylated base will be less likely to cause the loss of methyl groups than replication of the lesion followed by recombination.

### *Effects of ethionine, promotors and bromodeoxyuridine*

There is at least one carcinogen, ethionine, which would not be expected to alter the structure of DNA in any way. Ethionine is an analogue of methionine and its carcinogenic activity is restricted to liver tissue (Farber, 1963). It can be metabolized in liver to replace the normal amino acid in S-adenosyl methionine. S-adenosylethionine is a poor substrate for known methylating enzymes, since the normal process of DNA methylation is impaired (Swann *et al.*, 1975). It is, however, also possible that ethyl groups could be donated to DNA (Swann *et al.*, 1971) and these could inhibit the maintenance enzyme. The theory I have outlined can thus explain the carcinogenic activity of ethionine, because its presence in dividing cells could lead to the loss of methylation in controlling sequences. Recently it has been shown that ethionine can stimulate the *in vitro* differentiation of Friend cells (Christman *et al.*, 1977) and that this is related to a reduction in DNA methylation.

In many instances it has been shown that the effect of carcinogens is weak or absent unless promoting agents, such as phorbol esters, are subsequently applied. Moreover, the initial effect of the carcinogen is "remembered", since promotors are almost as effective when used many months after carcinogenic treatment as they are when used immediately (for a review see Berenblum, 1974). There is evidence that promotors can block terminal differentiation of mouse erythroleukaemia cells and chick myoblast cultures (Cohen *et al.*, 1977; Rovera *et al.*, 1977; Yamasaki *et al.*, 1977). If their action is related to the control of gene expression, they may well interact with cells in which abnormal switches in gene activity have already occurred. In particular, by preventing terminal differentiation, they might allow the continued proliferation of cancerous or pre-cancerous cells.

Bromodeoxyuridine (BUdR) also blocks cell differentiation in a variety of situations (for a review see Rutter *et al.*, 1973). Recently it has been found that human foetal lung fibroblasts grown in the presence of low levels of BUdR for many cell generations assume many of the properties of transformed cells, including loss of contact inhibition, growth in soft agar or low levels of serum (L. I. Huschtscha & R. Holliday, unpublished results). This phenotype persists when BUdR is removed. It is striking that all attempts to transform human foetal fibroblasts with potent mutagens have failed, whereas an agent known to effect cell differentiation may apparently induce transformation.

Kinsella & Radman (1978) have recently obtained evidence that promotors induce sister-strand recombination in mammalian cells. They suggest that their promoting action is due to the expression of recessive mutations by mitotic crossing over (see also Peto *et al.*, 1975). It is possible that mitotic crossing over between homologues plays a role in the mechanism we have proposed, since the switching off of a gene could be a recessive epigenetic event which must become homozygous to affect the phenotype. However, this recombination between homologues seems to be extremely rare or absent in mammalian somatic cells (Gladstone *et al.*, 1976; Tarrant & Holliday, 1977; Rosenstraus & Chasin, 1978).

### CONCLUSIONS

Holliday & Pugh (1975) have previously proposed a theory of development which attempted to provide a molecular basis for the segregation of gene activities during development and the very great stability of the differentiated state. It was suggested that specific controlling sequences adjacent to structural genes are modified by specific methylating enzymes, and that the presence or absence of such modification constitutes the "on" and "off" signals for transcription of the adjacent structural genes. Once the switch is set, the presence or absence of methylation in different cells is stably maintained, or heritable. This programming process, which is assumed to occur throughout the whole of development of complex organisms, is reversed at oogenesis or during the early cleavage divisions by the removal or dilution out of all methyl groups. I have now shown that damage to DNA which is susceptible to repair mechanisms could lead to the loss of specific DNA methylations, which in turn would lead to changes in gene expression. If the gene concerned is itself involved in setting the pattern of modified bases in differentiated cells, one might expect multiple effects on gene activity, including the appearance of embryonic antigens. These changes could occur over several cell generations, but the final phenotype of the cancer cells might be relatively stable, with the retention of some of the specialized properties of the cell type from which it was derived.

In outlining the theory, I have avoided discussion of viral oncogenesis. The integration of a viral genome could clearly change gene activity directly, as in mutation, or indirectly, as in this DNA-modification hypothesis. I am not aware of any studies on tumour viruses which either specifically support or are contrary to our model. Nor is it possible to make specific predictions about the malignancy of hybrids obtained by fusing malignant cells with non-malignant ones, since epigenetic changes could be either dominant or recessive. It might, however, be expected that the fusion of 2 different types of differentiated diploid cell to form a hybrid would sometimes have pleiotropic consequences comparable to those we have been discussing. This stems from the point raised earlier, namely, that developmental pathways diverge from each other and that specialized differentiated cells contain gene products (especially specific methylases) which are never normally in direct contact with those from other specialized cell types. Therefore abnormalities in gene expression might be expected. It is remarkable that in all hybridization experiments so far reported, either one of the parents is already a transformed line, or both parents are fibroblasts. The prediction that the fusion of two normal cells from different tissues might sometimes lead to a transformed phenotype has therefore not yet been experimentally tested. The theory also predicts that the very great difference in the frequency of carcinogenesis in rodents and large long-lived animals (Peto, 1977) is due to the activity of specific methylating enzymes and the efficiency of excision repair, rather than to differences in mutation frequency. I feel that the case for a mutational origin of cancer has often been overstated, perhaps because it has been too readily assumed that DNA damage is equivalent to mutation. Nevertheless, it is appropriate to conclude by stating that none of the above suggestions exclude the possibility that some tumours are the result of gene mutation.

I thank R. Peto, H. J. F. Cairns, P. D. Lawley and M. H. Green for helpful comments.

### REFERENCES

ADAMS, R. L. P. (1974) Newly synthesised DNA is not methylated. *Biochim. Biophys. Acta*, **335**, 365.

AMES, B. N., DURSTON, W. E., YAMASAKI, E. & LEE, F. D. (1973) Carcinogens are mutagens: a simple test system combining liver homogenates for activation and bacteria for detection. *Proc. Natl Acad. Sci., U.S.A.*, **70**, 2281.

BERENBLUM, I. (1974) *Carcinogenesis is a Biological Problem*. Oxford: North-Holland.

CAIRNS, H. J. F. (1978) *Cancer: Science and Society*. San Francisco: W. H. Freeman.

CHRISTMAN, J. K., PRICE, P., PEDRINAN, L. & ACS, G. (1977) Correlation between hypomethylation of DNA and expression of globin genes in Friend erythroleukaemia cells. *Eur. J. Biochem.*, **81**, 53.

CLEAVER, J. E. & BOOTSMA, D. (1975) Xeroderma pigmentosum: biochemical and genetic characteristics. *Ann. Rev. Genet.*, **9**, 19.

COGGIN, J. H. & ANDERSON, N. G. (1974) Cancer, differentiation and embryonic antigens: some central problems. *Adv. Cancer Res.*, **19**, 106.

COHEN, R., PACIFICI, M., RUBENSTEIN, N., BIEHE, J. & HOLTZER, H. (1977) Effect of a tumour promotor on myogenesis. *Nature*, **266**, 538.

DE WEERD-KASTELEIN, E. A., KEIJZER, W., RAINALDI, G. and BOOTSMA, D. (1977) Induction of sister chromatid exchanges in Xeroderma pigmentosum cells after exposure to ultraviolet light. *Mutat. Res.*, **45**, 253.

FARBER, E. (1963) Ethionine carcinogenesis. *Adv. Cancer Res.*, **7**, 383.

FURTH, J. (1947) Neoplastic transformation of granulosa cells in grafts of normal ovaries into spleens of gonadectomized mice. *J. Natl Cancer Inst.*, **8**, 7.

GLADSTONE, P., SABO, K., PIOUS, L. & PIOUS, D. (1976) Correlation between production of quadriradial chromosome configurations and mitotic recombinants in the HLA region in cultured lymphoid cells. *Proc. Vth Int. Congr. Hum. Genet.*, p. 127.

HANAWALT, P. C., FRIEDBERG, E. C. & FOX, C. F. (Eds.) (1978) *DNA Repair Mechanisms*. New York: Academic Press.

HART, R. W. & SETLOW, R. B. (1974) Correlation between deoxyribonucleic acid excision-repair and lifespan in a number of mammalian species. *Proc. Natl Acad. Sci. U.S.A.*, **71**, 2169.

HOLLIDAY, R. (1964) A mechanism for gene conversion in fungi. *Genet. Res.*, **5**, 282.

HOLLIDAY, R. & PUGH, J. E. (1975) DNA modification mechanisms and gene activity during development. *Science*, **187**, 226.

HUBERMAN, E., MAGER, R. & SACHS, L. (1976) Mutagenesis and transformation of normal cells by chemical carcinogens. *Nature*, **264**, 360.

KAUFFMAN, S. A. (1973) Control circuits for determination and transdetermination. *Science*, **181**, 310.

KING, T. J. & DI BERARDINO, M. A. (1965) Transplantation of nuclei from the frog renal adenocarcinoma. I. Development of tumor nuclear transplant embryos. *Ann. Rev. N.Y. Acad. Sci.*, **126**, 115.

KINSELLA, A. R. & RADMAN, M. (1978) Tumor promotor induces sister chromatid exchanges: Relevance to mechanisms of carcinogenesis. *Proc. Natl Acad. Sci.*, **75**, 6149.

LAWLEY, P. D. (1979) Approaches to chemical dosimetry in mutagenesis and carcinogenesis: the relevance of reactions of chemical mutagens and carcinogens with DNA. In *Chemical Carcinogens and DNA*. Ed. P. L. Grover. Florida: CRC Press.

LAWLEY, P. D. & MARTIN, C. N. (1975) Molecular mechanisms in alkylation mutagenesis. Induced reversion of bacteriophage T4r11 AP72 by EMS in relation to extent of ethylation of purines in bacteriophage DNA. *Biochem. J.*, **145**, 85.

LEHMANN, A. R. (1972) Post replication repair of DNA in ultraviolet-irradiated mammalian cells. *J. Mol. Biol.*, **66**, 319.

LOVELESS, A. (1969) Possible relevance of 06 alkylation of deoxyguanosine to mutagenicity of nitrosamines and introsamides. *Nature*, **223**, 206.

MCCANN, J., CHOI, E., YAMASAKI, E. & AMES, B. N. (1975) Detection of carcinogens as mutagens in the Salmonella/microsome test: Assay of 300 chemicals. *Proc. Natl Acad. Sci., U.S.A.*, **72**, 5135.

MCCANN, J. & AMES, B. N. (1976) Detection of carcinogens as mutagens in the Salmonella/microsome test: Assay of 300 chemicals: Discussion. *Proc. Natl Acad. Sci. U.S.A.*, **73**, 950.

MCKINNELL, R. G., DEGGINS, B. A. & LABAT, D. D. (1969) Transplantation of pluripotential nuclei from triploid frog tumours. *Science*, **165**, 394.

Markert, C. L. (1968) Neoplasia: A disease of cell differentiation. *Cancer Res.*, **28**, 1908.

Marquardt, H. (1974) Cell cycle dependence of chemically induced malignant transformation *in vitro*. *Cancer Res.*, **34**, 1612.

Medawar, P. B. (1977) Anaplasia rediviva. *Ann. Intern. Med.*, **87**, 100.

Meneghini, R. & Hanawalt, P. (1976) T4-endonuclease V-sensitive sites in DNA from ultraviolet-irradiated human cells. *Biochim. Biophys. Acta*, **425**, 428.

Meneghini, R. & Menck, C. F. M. (1978) Pyrimidine dimers in DNA strands of mammalian cells synthesized after UV irradiation. In *DNA Repair Mechanisms*. Ed. P. C. Hanawalt, E. C. Friedberg & C. F. Fox. New York: Academic Press. p. 493.

Meselson, M. & Radding, C. M. (1975) A general model for genetic recombination. *Proc. Natl. Acad. Sci. U.S.A.*, **72**, 358.

Mintz, B. & Illmensee, K. (1975) Normal genetically mosaic mice produced from malignant teratocarcinoma cells. *Proc. Natl Acad. Sci., U.S.A.*. **72**, 3585.

Moore, P. D. & Holliday, R. (1976) Evidence for the formation of hybrid DNA during mitotic recombination in Chinese hamster cells. *Cell*, **8**, 573.

Papaionnou, V. E., McBurney, M. W., Gardner, R. L. & Evans, M. J. (1975) Fate of teratocarcinoma cells injected into early mouse embryos. *Nature*, **258**, 70.

Pegg, A. E. (1977) Formation and metabolism of alkylated nucleosides: possible role in carcinogenesis by nitroso compounds and alkylating agents. *Adv. Cancer Res.*, **25**, 195.

Perry, P. & Evans, H. J. (1975) Cytological detection of mutagen-carcinogen exposure by sister chromatid exchange. *Nature*, **248**, 121.

Peto, R., Roe, F. J. C., Lee, P. N., Levy, L. & Clack, J. (1975) Cancer and ageing in mice and men. *Brit. J. Cancer*, **32**, 411.

Peto, R. (1977) Epidemiology, multistage models and short-term mutagenicity tests. In *Origins of Human Cancer*, Ed. Hiatt, Watson & Winsten. Cold Spring Harbor Laboratory. p. 1403.

Potter, H. & Dressler, D. (1976) On the mechanism of genetic recombination: electron microscope observation of recombination intermediates. *Proc. Natl Acad. Sci. U.S.A.*, **73**, 3000.

Pugh, J. E. & Holliday, R. (1978) Do chemical carcinogens act by altering epigenetic controls through DNA repair rather than by mutations? *Heredity*, **40**, 329.

Rommelaere, J. & Miller-Faures. (1975) Detection by density equilibrium centrifugation of recombinant-like DNA molecules in somatic mammalian cells. *J. Mol. Biol.*, **98**, 195.

Rosenstraus, M. J. & Chasin, L. A. (1978) Separation of linked markers in Chinese hamster cell hybrids: mitotic recombination is not involved. *Genetics*, **90**, 735.

Roth, R. & Rajewsky, M. F. (1974*a*) Molecular and cellular mechanisms associated with pulse-carcinogenesis in the rat nervous system by ethylnitrosourea: ethylation of nucleic acids and elimination rates of ethylated bases from the DNA of different tissues. *Z. Krebsforsch.*, **82**, 37.

Roth, R. & Rajewsky, M. F. (1974*b*) Persistence of 06-ethyl guanine in rat brain DNA: Correlation with nervous system-specific carcinogenesis by ethylnitrosamine. *Proc. Natl Acad. Sci. U.S.A.*, **71**, 639.

Rovera, G., O'Brien, T. A. & Diamond, L. (1977) Tumor promotors inhibit spontaneous differentiation of Friend erythroleukaemia cells in culture. *Proc. Natl Acad. Sci.*, **74**, 2894.

Rupp, W. D., Wilde C. E. III, Reno, D. L. & Howard-Flanders, P. (1971) Exchanges between DNA strands in ultraviolet irradiated *Escherichia coli*. *J. Mol. Biol.*, **61**, 25.

Rutter, W. J., Pictet, R. L. & Morris, P. W. (1973) Toward molecular mechanisms of developmental processes. *Ann. Rev. Biochem.*, **42**, 601.

Salas, C. E., Pfohl-Leszkowicz, A., Lang, M. C. & Dirheriner, G. (1979) Effect of modification by N-acetoxy-N-2 acetylaminofluorene on the level of DNA methylation. *Nature*, **278**, 71.

Scarano, E. (1971) The control of gene function in cell differentiation and in embryogenesis. *Adv. Cytopharmacol.*, **1**, 13.

Sigal, N. & Alberts, B. (1972) Genetic recombination: the nature of a cross-strand exchange between two homologous DNA molecules. *J. Mol. Biol.*, **71**, 789.

Sobell, H. M. (1974) Concerning the stereochemistry of strand equivalence in genetic recombination. In *Mechanisms in Recombination*, Ed. R. F. Grell. New York: Plenum Press. p. 433.

Swann, P. F., Pegg, A. E., Hawks, A., Farber, E. & Magee, P. N. (1971) Evidence for ethylation of rat liver deoxyribonucleic acid after administration of ethionine. *Biochem. J.*, **123**, 175.

Swann, P. F., Peacock, A. C. & Bunting, S. (1975) Carcinogenesis and cellular injury. The effect of ethionine on ribonucleic acid synthesis in rat liver. *Biochem. J.*, **150**, 335.

Tarrant, G. M. & Holliday, R. (1977) A search for allelic recombination in Chinese hamster cell hybrids. *Mol. Gen. Genet.*, **156**, 273.

Venner, H. & Reinert, H. (1973) Possible role of methylated DNA bases for the transcription of genetic information. *Z. Allg. Mikrobiol.*, **13**, 613.

Yamasaki, H., Fibach, E., Nudel, U., Weinstein, I. B., Rijkund, R. A. & Marks, P. A. (1977) Tumor promotors inhibit spontaneous and induced differentiation of murine erythroleukaemia cells in culture. *Proc. Natl Acad. Sci., U.S.A.*, **74**, 3451.